# Facial feedback effect on the sense of body ownership during the rubber hand illusion

**DOI:** 10.3389/fnhum.2023.976290

**Published:** 2023-03-15

**Authors:** Yoshitaka Kaneno, Hiroshi Ashida

**Affiliations:** Department of Psychology, Graduate School of Letters, Kyoto University, Kyoto, Japan

**Keywords:** rubber hand illusion, body ownership, facial feedback hypothesis, emotion, multisensory integration

## Abstract

The sense of body ownership, a feeling that one’s body belongs to the self, is an essential aspect of self-consciousness. Studies have focused on emotions and bodily states that could influence multisensory integration for the sense of body ownership. Based on the Facial Feedback Hypothesis, the purpose of this study was to examine whether displaying specific facial expressions affects the rubber hand illusion. We hypothesized that the expression of a smiling face changes the emotional experience and facilitates the formation of a sense of body ownership. In the experiment, participants (*n* = 30) were asked to hold a wooden chopstick in their mouths to simulate smile, neutral, and disgusted facial expressions during the induction of the rubber hand illusion. The results did not support the hypothesis, showing that proprioceptive drift (an index of illusory experience) was enhanced when the subjects displayed a disgusted facial expression, while the subjective reports of the illusion were not affected. These results, together with the previous studies regarding the effect of positive emotions, suggest that bodily affective information, regardless of its valence, facilitates multisensory integration and could influence the conscious representation of the bodily self.

## 1. Introduction

One immediately knows that one’s own body is feeling sensory stimuli, such as wind blowing against one’s cheek, even if one is not explicitly aware of it. The feeling that parts of one’s body belong to the whole body is called *sense of body ownership* (Gallagher, 2000; Ehrsson, 2020). Sense of body ownership is a basis of self-consciousness, which is essential to survival and adaptive action in the ever-changing environment (Ehrsson et al., 2004).

Several studies investigating the sense of body ownership have used the *Rubber Hand Illusion* (Botvinick and Cohen, 1998), an experimental paradigm that creates an illusory experience of artificial limbs being a part of one’s body representation. In the rubber hand illusion paradigm, an experimenter simultaneously presents both a participant’s hand and a fake hand with tactile stimuli typically using a brush, and gradually the participants feel as if the fake hand belonged to their body. This paradigm has made it possible to explore how the sense of body ownership is constructed. Previous research has shown that the sense of body ownership is not only the result of the bottom-up process of multisensory integration, but also is modulated by top-down effects of cognition and emotion (Kilteni et al., 2015).

Recent studies have increasingly focused on the contribution of emotion to forming the body ownership, which is in line with psychological and neurocognitive results suggesting that affective information can alter our perception, cognition, and other post-perceptual processes (Zadra and Clore, 2011; Niedenthal and Wood, 2019). For example, participants tended to feel stronger rubber hand illusion, when presented with visual stimuli that elicit specific emotions such as awe (Takano and Nomura, 2021) and sadness (Schroter et al., 2021). Affective touch, which is slow and caress-like touch that yields pleasant feelings, modulates the sense of body ownership and induces stronger rubber hand illusion (Crucianelli et al., 2013, 2018; van Stralen et al., 2014). van Stralen et al. (2014) suggested that interoceptive induction of positively-valenced emotions by affective touch might function as a cue that facilitates multisensory integration of conflicting sensory inputs, which results in modulation of the sense of body ownership. These studies suggest a link between emotional states and body representation; specifically, positively-valenced interoceptive information (such as pleasantness induced by affective touch) might facilitate the sense of body ownership (Tsakiris et al., 2011; Seth, 2013; Suzuki et al., 2013).

Facial muscular movements can influence emotional states. The growing literature has suggested that feedback information from facial movements (such as raising the cheeks with a pen) can in turn be crystallized into a specific emotion or can influence the perception of emotionally valenced stimuli *via* interoceptive changes, which is called *Facial Feedback Hypothesis* (Strack et al., 1988; Zajonc et al., 1989; Izard, 1990; Coles et al., 2019; Marmolejo-Ramos et al., 2020). It has been investigated whether simple manipulation of facial muscle activity by holding a pen in a mouth (Strack et al., 1988) can modulate the ongoing emotional feelings and affective judgments on stimuli (Niedenthal, 2007; Hyniewska and Sato, 2015; Coles et al., 2019; Yu and Kitayama, 2021). Despite much controversy (Wagenmakers et al., 2016; Hiraishi and Nakamura, 2022), facial feedback is found to affect emotional experience (especially happiness, see Coles et al., 2022) weakly but robustly (Noah et al., 2018; Marsh et al., 2019; Yu and Kitayama, 2021). In addition, information from facial nerves is integrated with interoceptive information (Kandel et al., 2013). In the case of the rubber hand illusion, the facial feedback information might, by an analogy of affective touch, affect pleasantness of the illusion-inducing stimulus, which might then modulate the sense of body ownership and thus the strength of the illusion. Little is known, however, about the effect of facial muscular movements on the sense of body ownership.

The present study investigated whether and to what extent the facial feedback procedure affects the rubber hand illusion. We hypothesized that displaying a smiling facial expression elicits a stronger illusory experience during the rubber hand illusion by modulating tactile pleasantness, compared to other facial expressions including neutral face and disgust. To this end, we used the classical rubber hand illusion paradigm and measured subjective (questionnaire) and behavioral (proprioceptive drift) indices of the illusory experience *via* a questionnaire and a pointing task. During the induction of the illusion, we asked the participants to hold a stick in their mouth, covertly forming a specific facial expression (Strack et al., 1988; Ueda et al., 2017; Yu and Kitayama, 2021).

## 2. Methods

### 2.1. Participants

A total of 30 volunteers participated in the experiment (16 males and 14 females; Mean age = 26.07 years, SD = 4.06 years, 27 right-handed, three left-handed). Post-hoc power analysis (G*Power 3.1, Faul et al., 2007, 2009) revealed that the power (1-β) was >0.95, based on α = 0.05 and medium effect size ($\eta_{p}^{2}$ = 0.1). All participants had a normal or corrected-to-normal vision and were naïve to the research hypothesis. Written informed consents were obtained from all participants. The experimental protocol was approved by the ethics committee of Kyoto University Psychological Science Unit (Approval number: 3-P-24).

### 2.2. Apparatus and stimuli

**FIGURE 1:**
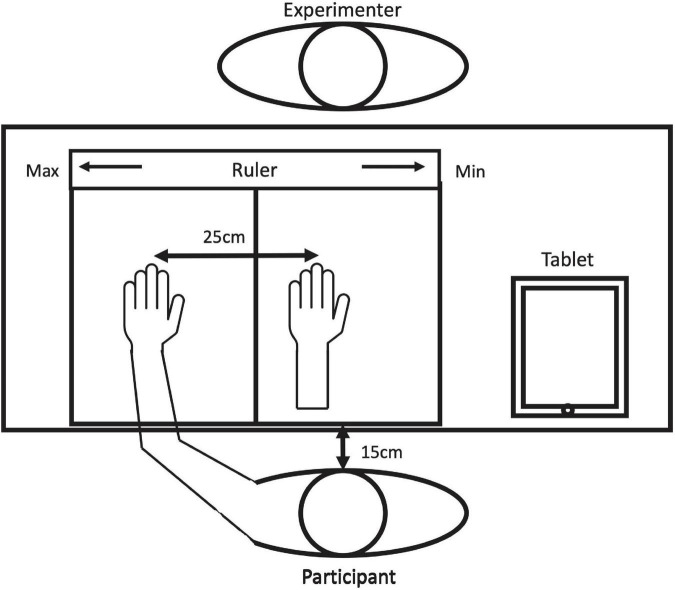
Schematic illustration of the experimental setup. The ruler was attached to the experimenter’s side of the box, with its scale larger toward the right from the experimenter.

Participants were seated confronting a polystyrene foam box (width: 65 cm × depth: 44 cm × heights: 34 cm). The box was separated at the center to accommodate the participant’s left hand on the left side and the fake hand on the right side. The top of the right side was open so that the participants could see the fake hand. The left side was closed, and a black cloth prevented the participant from seeing his or her hand. The box was placed approximately 15 cm away from the participant so that the center of the box was aligned with the edge of the participant’s left shoulder. There was approximately 25 cm distance between the participant’s hand and the fake hand. A ruler was attached to the experimenter side to measure the participants’ responses (see Figure 1).

**FIGURE 2:**
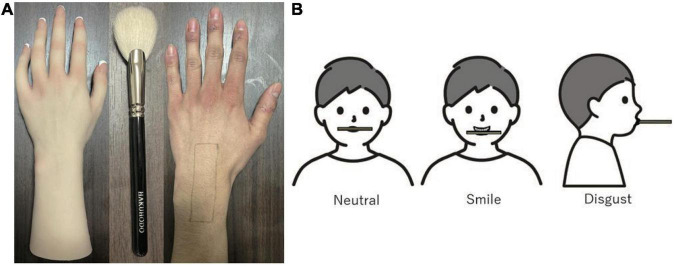
**(A)** The fake hand (left) and the brush (middle) used in the experiment, and the area on the real hand for the presentation of tactile stimuli (right). **(B)** Facial manipulations with a wooden stick.

A silicone human-like hand was used as a fake hand (Figure 2A left), and tactile stimuli were applied to the participant’s hand and the fake hand with two identical cosmetic brushes (Hakuho-Do, goat hair and synthetic fiber, hair length: 28 mm) (Figure 2A center). Tactile stimuli were presented by a trained experimenter to the marked area of the participant’s hand (9 cm sagittally × 2 cm coronally, see Figure 2A right) at 1 Hz for 60 s. In the Synchronous condition, tactile stimuli were presented simultaneously to the participant’s hand and the fake hand, whereas in the Asynchronous condition tactile stimuli were presented first to the fake hand, then to the participant’s hand after the short delay (approximately 500 ms).

### 2.3. Facial feedback procedure

Participants were asked to hold a wooden chopstick in their mouths so that they express three different facial expressions in each trial before the induction of the illusion (Figure 2B). Participants held a wooden chopstick horizontally by their lips in the center (Neutral condition), by their back teeth (Smile condition), or by their rounded lips with their mouth closed and the stick pointing forward (Disgust condition). Participants were not directly instructed to express a specific emotion. The order of facial expressions was randomized.

### 2.4. Measurements

#### 2.4.1. Proprioceptive drift

We used proprioceptive drift (PD) as a behavioral measure of the illusory experience, which is a phenomenon in which the perceived position of the real hand is shifted toward the fake hand and is commonly used in the studies of rubber hand illusion. Participants closed their eyes and pointed to the perceived location of their left index fingertips with their right index finger on the top surface of the box, before (Pre-PD) and after (Post-PD) the tactile stimulation in each trial. The PD index was defined as the difference between the Post-PD and Pre-PD values, with a positive value indicating a shift toward the fake hand (to the right). A larger positive PD index, therefore, indicates a stronger rubber hand illusion.

#### 2.4.2. Questionnaire on the rubber hand illusion

For an index of the subjective experience of the rubber hand illusion, we asked participants to answer a questionnaire in Japanese (see Supplementary Table 1), translated from previous studies (Botvinick and Cohen, 1998; van Stralen et al., 2014; see Supplementary Table 2). The questions were created using Lab.js (Henninger et al., 2021) and were presented on a tablet device (iPad 5th generation, Apple Inc., Cupertino, CA, USA). The participants responded by adjusting the slider on the touchpad with their right hands.

Participants responded to each item on a Likert scale of 0–10 (0: “totally disagree,” 5: “neither agree nor disagree,” 10: “totally agree”) regarding the degree of the illusion in each trial. The order of questionnaire items was randomized on a trial-by-trial basis. The items 1–3 were regarded as illusion-related, whereas the items 4–10 were as control (van Stralen et al., 2014). We analyzed each score of the questionnaire items as an index of subjective illusory experience, following van Stralen et al. (2014).

#### 2.4.3. Rating of pleasantness

To assess the effect of facial expression feedback, participants rated how pleasant the tactile stimulus was on a discrete scale of 0–100 (0: “not pleasant at all” to 100: “very pleasant”) by moving the slider presented on the tablet device. The standardized score (z-score) of the rating was used for analysis. The slider started from a default value of 50, and the participants could not see the actual values of their pleasantness rating.

### 2.5. General procedure

Before the experiment, participants were given instructions on the rubber hand illusion procedures. They were also instructed to attend to the fake hand during the illusion induction. During the experiment, they rested their left forearms in the left half of the box with their palm down on the table.

In each trial, first, the participants closed their eyes and made the Pre-PD response. Then, they held a stick to form one of the facial expressions, and the tactile stimulus was presented for 60 s (either synchronous or asynchronous), followed by the Post-PD response. After that, they answered the questionnaire (10 items) and then made the pleasantness rating with the tablet device.

Each participant conducted a total of 6 trials of different conditions (3 facial expressions × 2 visuo-tactile stimulation synchrony), in a randomized order.

Participants took a short break between trials, closing their eyes and holding or opening their palms without changing the position of their left hand. The experimenter provided a briefing regarding the research hypothesis at the end of the experiment.

### 2.6. Analysis

The analysis was performed using R (ver. 4.0.4, R Core Team, 2022) and anovakun (ver. 4.8.6). Among the 30 participants, three were excluded from the analysis because of incomplete experimental procedures. Thus, the total number of participants in the analysis was 27 (14 males and 13 females; mean age = 24.22 years; 24 right-handed and three left-handed).

Dependent variables in the analysis were: (1) proprioceptive drift index, (2) illusion rating scores, and (3) standardized scores of the pleasantness rating, as described before. For proprioceptive drift and pleasantness, a two-way repeated-measure analysis of variance was conducted with facial expressions (smile, neutral, and disgust) and synchrony (synchronous and asynchronous) as within-participant factors. For the questionnaire scores, a Friedman’s test was conducted with facial expressions, synchrony, and questionnaire items (statement 1–10) as within-participant factors. For the post-hoc multiple comparisons, *t*-tests with Holm’s method of correction were used for proprioceptive drift index and pleasantness scores, and Wilcoxon’s signed rank tests with Benjamini-Hochberg method of controlling the false discovery rate for questionnaire scores (using p.adjust () function of R). Squared eta (η^2^) was used as an index of the effect size. Mendoza’s multisample sphericity test was conducted on each dependent variable. If the sphericity assumption is violated, we adjusted the degree of freedom by the Greenhouse-Geisser’s epsilon.

## 3. Results

### 3.1. Proprioceptive drift

**FIGURE 3:**
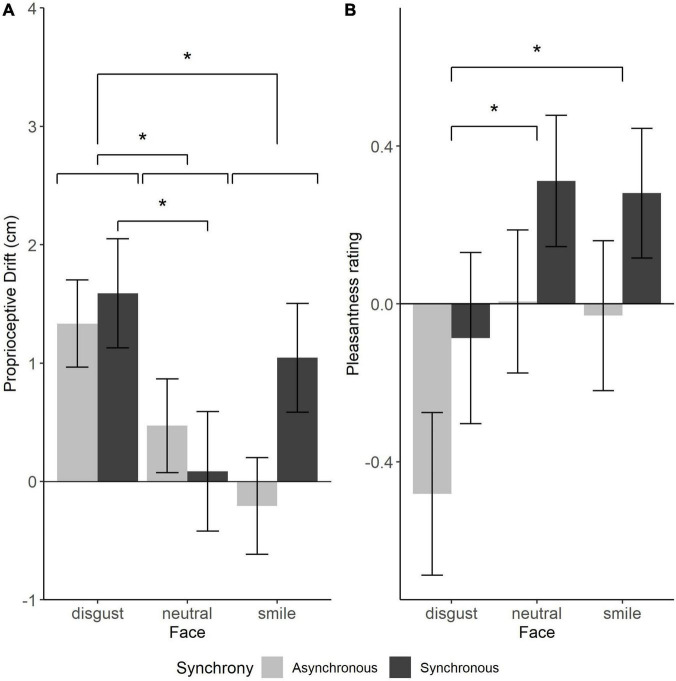
**(A)** Proprioceptive drift (cm). **(B)** Standard scores of pleasantness. Dark and light bars represent synchronous and asynchronous stroking conditions, respectively, averaged across participants. Error bars show standard errors of mean. *p < 0.05.

The mean PD indices for each condition are shown in Figure 3A. Sphericity indices showed no violation of the sphericity assumption. A repeated-measure ANOVA showed a significant effect of the facial expression [*F* (2, 52) = 6.01, *p* = 0.005, η^2^ = 0.052, 95% CI = (0.005, 0.107) by bootstrapping]. Post-hoc analysis revealed that the disgust expression yielded larger drift than the other two [vs. smiling: *t* (26) = 3.05, adjusted *p* = 0.016; vs. neutral: *t* (26) = 2.95, adjusted *p* = 0.007]. The main effect of synchrony or the interaction between facial expression and synchrony was not significant [synchrony: *F* (2, 52) = 1.02, *p* = 0.323, η^2^ = 0.007, 95% CI = (0.000, 0.060); interaction: *F* (2, 52) = 2.62, *p* = 0.083, η^2^ = 0.021, 95% CI = (0.001, 0.055)].

### 3.2. Questionnaire

**FIGURE 4:**
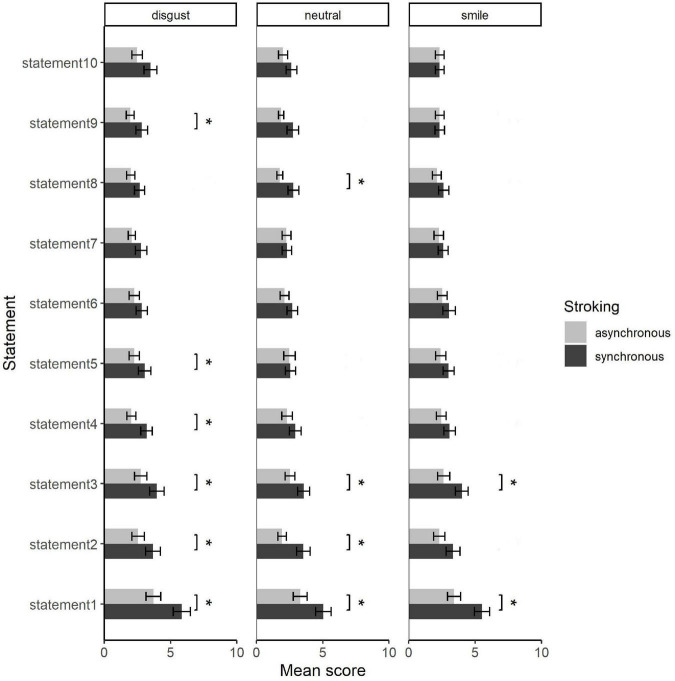
Rating scores of each statement for each condition averaged across participants. Error bars show standard errors of mean. *p < 0.05 with controlled false discovery rate.

Figure 4 shows the mean scores of each questionnaire for each condition. Friedman’s test revealed significant effect of synchrony [χ^2^ (1) = 50.96, *p* < 0.001] and questionnaire items [χ^2^ (9) = 150.16, *p* < 0.001], but not the effect of facial expressions [χ^2^ (2) = 4.32, *p* = 0.116]. Multiple comparison using Wilcoxon’s signed rank test revealed larger rating scores for synchronous than asynchronous stimulation for items 1–3 (illusion-related) under all facial conditions except the smile condition of item 2 (item1: all *z*s < –2.91, and all adjusted *p*s < 0.027; item2: all *z*s < –2.60, and all adjusted *p*s < 0.027; item3: all *z*s < –2.44, and all adjusted *p*s < 0.037), while control items partially showed no difference, at the false discovery rate of q < 0.05. Control items showed this difference only for limited conditions (items 4, 5, 9 under the disgust: all *z*s < –2.46, and all adjusted *p*s < 0.037; and item 8 under the neutral condition: *z* = –2.64, adjusted *p* = 0.027). The effect of synchrony is in line with the previous studies, confirming that rubber hand illusion was properly induced. Questionnaire item 1 (illusion-related) with synchronous stimulation was rated above 5 (neutral) on average, while all control items were rated below 5, also confirming the induction of illusion. However, other illusion-related items (2 and 3) were rated below neutral, suggesting that the illusion might have been relatively weak.

### 3.3. Pleasantness

The standard scores (z-scores) of the pleasantness rating for each condition are shown in Figure 3B. Sphericity indices showed no violation of the sphericity assumption. A repeated-measure ANOVA revealed significant main effects of facial expression [*F* (2, 52) = 6.45, *p* = 0.003, η^2^ = 0.041, 95% CI = (0.003, 0.110)] and synchrony [*F* (1, 26) = 5.26, *p* = 0.003, η^2^ = 0.029, 95% CI = (0.002, 0.105)]. Post-hoc analysis using the Holm’s method showed that pleasantness ratings were significantly lower for disgust expression compared to both neutral and smiling expressions [neutral: *t* (26) = 2.69, adjusted *p* = 0.037; smiling: *t* (26) = 2.63, adjusted *p* = 0.037]. The participants rated the tactile stimuli as less pleasant when they formed disgusted facial expression, and as more pleasant when they formed smiling and neutral expressions. However, there was no significant difference between the smiling and neutral conditions [*t* (26) = 0.46, adjusted *p* = 0.65]. There was no significant interaction between facial expression and synchrony [*F* (2, 52) = 0.09, *p* = 0.907, η^2^ = 0.000, 95% CI = (0.000, 0.002)].

## 4. Discussion

The present study aimed to investigate the effect of facial feedback information on the sense of body ownership. To this end, we used the conventional rubber hand illusion paradigm and covertly manipulated the participant’s facial expressions with a stick. We expected that participants displaying smiling expressions experience a stronger illusion than when displaying disgusted or neutral expressions. This expectation, however, was not supported. We rather found that forming a disgusted expression elicited larger proprioceptive drift during the rubber hand illusion than smiling or neutral expressions. Moreover, self-generated expressions did not affect the subjective illusory experience as measured by the questionnaire, while the illusion was induced as synchronous stimulation evoked a stronger illusory experience than asynchronous stimulation. These results suggest that the processing of bodily information has modulatory effects on the different aspects of the sense of body ownership, supporting the idea that proprioceptive drift and subjective reports of the rubber hand illusion depend on different information processing (Serino et al., 2013; Gallagher et al., 2021).

The main finding of this study was that disgusted facial expressions led to less pleasantness rating and larger proprioceptive drift. This result does not support our hypothesis that positive expressions enhance the rubber hand illusion, but is consistent with some earlier reports; the illusory experience is robustly enhanced in the presence of negatively-valenced stimuli such as a fearful spider (Chouinard and Stewart, 2020) and disgusting visual stimuli (Jalal et al., 2015; Nitta et al., 2018), by evoking sadness emotions with pictures (Schroter et al., 2021), and by the presentation of angry sounds (Engelen et al., 2017). The feedback effect of forming a disgusted facial expression might be explained by accompanying bodily changes such as the late positive potential (LPP) that implies emotional arousal (Yu and Kitayama, 2021), or by interoceptive changes in vascular blood flow to the brain (Zajonc et al., 1989). These affective and interoceptive cues could be integrated with visuo-tactile information, resulting in mislocation of a body part.

A possible explanation of the discrepancy between the results of the questionnaire and proprioceptive drift is that somatosensory processing of facial muscular movements is diminished to solve conflicting multisensory inputs during the rubber hand illusion (Isayama et al., 2019; Rossi Sebastiano et al., 2021). Thus, the offline representation of the body is not disturbed by bodily information, resulting in the robustness of the embodiment scores irrespective of the facial manipulation. Proprioceptive Drift, on the other hand, is considered to reflect the more direct multisensory integration of online sensory cues (Gallagher et al., 2021), which suggests that the localization of the bodily self is affected by the online bodily information. In the present study, the participants continuously formed a specific facial expression, which could serve as an ongoing affective cue and thus facilitated the multisensory integration process. Together with previous findings of the effect of tactile pleasantness (positively valenced stimuli) on the illusional experience (Lloyd et al., 2013; van Stralen et al., 2014; Crucianelli et al., 2018), it is suggested that bodily affective information, whether it is positive or negative, could affect the sense of body ownership. Engelen et al. (2017) actually demonstrated larger proprioceptive drift toward the fake hand when provided with an affective voice (angry or happy vocal stimuli) during the induction of the illusion. As most research focused on a single valence of emotions (awe, Takano and Nomura, 2021; sadness, Schroter et al., 2021; fear; Chouinard and Stewart, 2020), it remains an open question how positive and negative expressions can affect the sense of body ownership and the rubber hand illusion.

There is a caveat that the participants’ experience of illusion may not have been strong, as the overall scores of the questionnaire were relatively low. The low scores, however, might be explained by the tendency of the Asian population to avoid answering both extremes of Likert scale (Lee et al., 2002; Wang et al., 2008) with possibly stronger reservations for higher than lower scores that imply physically implausible events. Therefore, a fixed threshold of “neutral” may not be always adequate. We found clear differences between the scores under synchronous and asynchronous conditions that have been considered as a signature of the illusion, although Kalckert et al. (2019) have pointed out the problem of inference based solely on such a comparison. Our results include at least one item of the questionnaire that showed the score of synchronous condition above neutral. In addition, we found differences in proprioceptive drift as another indicator of the illusion, which was not discussed in Kalckert et al. (2019). Significant differences in some of the control items suggest that the control items somewhat include illusion-related constructs, while the scores are lower than those of the illusion-related items. Such a tendency was also seen in van Stralen et al. (2014). We therefore believe the questionnaire nevertheless captured the subjective experience of the rubber hand illusion. Another concern is that the demand of the facial task and/or the experimenter’s unconscious expectation effect in manipulation may have influenced participants’ responses. Such effects, however, should have led to the difference between the smiling and neutral conditions, which was not the case. In sum, our manipulation induced sufficient, if not optimal, illusory experience that was modulated by the facial feedback of disgust.

## 5. Conclusion

We found that, in the rubber hand illusion, the facial expressions of disgust elicited larger proprioceptive drift, while facial expressions did not modulate the subjective experience of the illusion. These findings suggest that bodily affective information, irrespective of its valence, modulates ongoing multisensory integration of sensory cues, although the effect may not be strong enough to affect the conscious representation of the bodily self. The present study adds to our understanding of the effect of bodily affective information on the sense of body ownership and might indirectly support the facial feedback hypothesis.

## Data availability statement

The raw data that support the findings of the study are available in the open science framework repository (https://osf.io/63p8t/). The code written in R 4.0.3 is also available in the above link.

## Ethics statement

The studies involving human participants were reviewed and approved by the Ethics Committee of Kyoto University Psychological Science Unit. The patients/participants provided their written informed consent to participate in this study.

## Author contributions

YK developed the study concept, performed the data collection, statistical analysis, and wrote the first draft of the manuscript. HA supervised these processes. Both authors contributed to the writing, design of the study, and approved the final version of the manuscript submission.
