# Supplementary material for: Facial feedback effect on the sense of body ownership during the rubber hand illusion
Source: Front Hum Neurosci. 2023 Mar 15;17:976290. doi: 10.3389/fnhum.2023.976290 (PMC10050436; doi:10.3389/fnhum.2023.976290)
Supplement: Supplementary file 1 [file Data_Sheet_1.docx]

Supplementary Material

Facial feedback effect on the sense of body ownership
during the rubber hand illusion

The Future of Perceptual Illusions: From Phenomenology to Neuroscience Vol II

***Front. Hum. Neurosci.* 17:976290.** [**doi: 10.3389/fnhum.2023.976290**](https://doi.org/10.3389/fnhum.2023.976290)

Yoshitaka Kaneno^*^ and Hiroshi Ashida

Department of Psychology, Graduate School of Letters, Kyoto University, Kyoto, Japan

# Supplementary Figures and Tables

Table 1. The Japanese questionnaire translated from van Stralen et al. (2014).

| **1** | ゴムの手が触れられている場所が実際に触られている気がした。 |
| --- | --- |
| **2** | 私が感じた触覚はゴムの手に与えられた刺激によるものだ。 |
| **3** | まるでゴムの手が自分自身の手のように感じられた。 |
| **4** | 私自身の手がゴムの手の方へ動いていくかのように感じた。 |
| **5** | 手や腕が2本以上あるかのような気がした。 |
| **6** | 私が感じていた触覚は私自身の手とゴムの手の間のどこかから来ていた。 |
| **7** | 私自身の手がゴムになっていくような気がした。 |
| **8** | ゴムの手が私自身の手の方へ動いてくるように見えた。 |
| **9** | ゴムの手が、形状や肌の色、そばかすなど見た目が私自身の手に似始めた。 |
| **10** | ゴムの手と私の利き手が互いに近寄っていくかのように感じた。 |

**Table 2**.

The original questionnaire (van Stralen et al., 2014, reproduced with permission.)

| Instructions:  Please keep the last trial in mind when rating the following questions according to a 1-10 scale (‘1’ means ‘I strongly disagree’, ‘5’ means ‘neutral’, and ‘10’ means ‘I strongly agree’).  During the last trial there were times when: | |
| --- | --- |
| 1 | I was feeling the touch at the location where I saw the rubber hand being touched. |
| 2 | The touch I felt was caused by the stimulation on the rubber hand. |
| 3 | I felt as if the rubber hand was my own hand. |
| 4 | It felt as if my real hand was drifting toward the rubber hand. |
| 5 | It felt as if I had more than two hands or arms. |
| 6 | It seemed as if the touch I was feeling came from somewhere between my own hand and the rubber hand. |
| 7 | It felt as if my real hand was turning ‘rubbery’. |
| 8 | It appeared (visually) as if the rubber hand was drifting toward my own hand. |
| 9 | The rubber hand began to resemble my own (real) hand, in terms of shape, skin tone, freckles or some other visual feature. |
| 10 | It felt as if the rubber hand and my own dominant hand lay closer to each other. |

# Reference

van Stralen, H. E., van Zandvoort, M. J., Hoppenbrouwers, S. S., Vissers, L. M., Kappelle, L. J., & Dijkerman, H. C. (2014). Affective touch modulates the rubber hand illusion. *Cognition*, *131*(1), 147-158.
